# Divergent Pro-Inflammatory Profile of Human Dendritic Cells in Response to Commensal and Pathogenic Bacteria Associated with the Airway Microbiota

**DOI:** 10.1371/journal.pone.0031976

**Published:** 2012-02-21

**Authors:** Jeppe Madura Larsen, Daniel Bisgaard Steen-Jensen, Janne Marie Laursen, Jonas Nørskov Søndergaard, Hanieh Sadat Musavian, Tariq Mahmood Butt, Susanne Brix

**Affiliations:** Systems Biology of Immune Regulation, Department of Systems Biology, Center for Biological Sequence Analysis, Technical University of Denmark, Lyngby, Denmark; National Jewish Health, United States of America

## Abstract

Recent studies using culture-independent methods have characterized the human airway microbiota and report microbial communities distinct from other body sites. Changes in these airway bacterial communities appear to be associated with inflammatory lung disease, yet the pro-inflammatory properties of individual bacterial species are unknown. In this study, we compared the immune stimulatory capacity on human monocyte-derived dendritic cells (DCs) of selected airway commensal and pathogenic bacteria predominantly associated with lungs of asthma or COPD patients (pathogenic *Haemophillus* spp. and *Moraxella* spp.), healthy lungs (commensal *Prevotella* spp.) or both (commensal *Veillonella* spp. and *Actinomyces* spp.). All bacteria were found to induce activation of DCs as demonstrated by similar induction of CD83, CD40 and CD86 surface expression. However, asthma and COPD-associated pathogenic bacteria provoked a 3–5 fold higher production of IL-23, IL-12p70 and IL-10 cytokines compared to the commensal bacteria. Based on the differential cytokine production profiles, the studied airway bacteria could be segregated into three groups (*Haemophilus* spp. and *Moraxella* spp. vs. *Prevotella* spp. and *Veillonella* spp. vs. *Actinomyces* spp.) reflecting their pro-inflammatory effects on DCs. Co-culture experiments found that *Prevotella* spp. were able to reduce *Haemophillus influenzae*-induced IL-12p70 in DCs, whereas no effect was observed on IL-23 and IL-10 production. This study demonstrates intrinsic differences in DC stimulating properties of bacteria associated with the airway microbiota.

## Introduction

The human body is host to an immense variety of bacterial species living in microbial communities (microbiotas) which may continuously change dependent on acquisition of bacteria encountered in the environment and clearance mediated by the immune system. It is becoming increasingly apparent that the colonizing bacteria are not merely invasive health treats giving rise to infections, but are symbiotes contributing to normal bodily functions through common mutualism [1]. Advances in high-throughput molecular biology have allowed in-depth characterization of microbiotas by abolishing the traditional laborious methods of bacteria identification by cultivation [2]. Instead, bacteria within microbial communities can be identified and quantified highly specific based on genetic composition. Due to the predictable importance of bacteria in the intestine, several studies have focused on characterizing the gut microbiota and addressing changes associated with disease, including autoimmune disease, allergy and obesity. Yet recent focus has turned to the less bacteria-laden mucosal surfaces of the airways, genitals, and skin, and diseases associated with these particular organs [3].

Healthy non-infected airways have historically been considered sterile due to the absence of identifiable bacteria using traditional methods of cultivation in most patients without clinically active bacterial pneumonia [4]–[6]. However, recent studies using molecular genetics have characterized the healthy human airway microbiota and identified colonization by several commensal and pathogenic bacteria of different phyla [7]–[10]. The microbiota of healthy airways was reported to consist predominantly of commensal bacteria from the bacteriodetes, firmicutes, proteobacteria and actinobacteria phyla [8]–[10]. Importantly, the airway microbiota was found to be significantly different from the gut microbiota indicating that microbial communities in the airways consist of bacteria adapted to live in conjunction with the conditions present in this organ [9]. When compared to healthy individuals, airways of asthmatics and COPD patients were recently reported to exhibit disordered microbial airway composition [7], [9]–[11]. Studies indicated that asthmatics and COPD patients were more commonly colonized with pathogenic proteobacteria (*Haemophilus* spp. and *Moraxella* spp.), whereas healthy individuals are more commonly colonized with commensal bacteriodetes (*Prevotella* spp.) [10]. Asthma and COPD are diseases giving rise to airway inflammation, and since airway bacteria are likely to interact with the immune system, it is of interest to study the immune stimulating properties of airway-colonizing bacteria to increase our general insight into bacteria-induced immunity. Furthermore, comparing properties between commensal and pathogenic strains of the airway microbiota may contribute to our understanding of disordered microbial communities observed in airway disease.

Dendritic cells are professional antigen presenting cells that play a central role in bridging innate and adaptive immunity by instructing the development of antigen-specific T cell responses [12]. In the present study we used a well-established model of human monocyte-derived dendritic cells [13], [14] to examine the immune stimulating properties of three pathogenic and six commensal bacteria associated with the airway microbiota. The studied airway bacteria could be segregated into three distinct groups based on the profile of cytokines produced by DCs. We propose that the distinct innate DC responses elicited by airway bacteria may be similar to those reported for commensal and pathogenic bacteria of the gastro-intestinal tract. Thus, commensal bacteria of the airways may in a similar manner play a role in maintaining immune homeostasis and controlling overt airway inflammation.

## Results

We selected three pathogenic and six commensal strains associated with healthy and diseased airway microbiotas (Table 1) for a comparative analysis. The pathogenic proteobacteria associated with asthma and COPD were *Haemophilus influenza* and *Moraxella* spp. (subspecies unknown) [10]. We included *Haemophilus influenza* B (H. inf. B) and non-typeable *Haemophilus influenza* (H. inf. NT) as both are common airway pathogens giving rise to infections, but structurally different as non-typeable *Haemophilus influenza* has no capsule and is predominantly associated with the respiratory tract [15], [16]. *Moraxella catarrhalis* (M. cat.) was included as the most common pathogenic *Moraxella* strain [17]. The commensal bacteroidetes *Prevotella melaninogenica* (P. mel.), *Prevotella nanceiensis* (P. nan.) and *Prevotella salivae* (P. sal.) were studied as representatives of bacteria associated with healthy airways [10]. The firmicutes *Veillonella dispar* (V. disp.) was enrolled as it is the most predominant airway commensal present in both healthy and diseased airways [10]. Lastly, commensal *Actinomyces graevenitzii* (A. grae.) and *Actinomyces oris* (A. oris.) were included as less common members of the actinobacteria phylum associated with both healthy and diseased airways [10]. All the bacteria designated as commensal are rarely reported to cause infectious disease compared to the common pathogenic bacteria and are here thus considered as commensals.

**Table 1:** Phyla and features of the bacterial strains analyzed.

Bacterial strain	Phylum	Gram	Feature	Ref.
*Haemophilus influenzae B*	Proteobacteria	Neg	Pathogenic bacteria found in the airway microbiota of asthma and COPD patients. Associated with development of asthma in children.	[10], [59]
*Haemophilus influenzae NT* *	Proteobacteria	Neg	Pathogenic bacteria present in the airway microbiota of asthma and COPD patients. Associated with development of asthma in children.	[10], [59]
*Moraxella catarrhalis*	Proteobacteria	Neg	Pathogenic bacteria found in the airway microbiota of asthma and COPD patients. Associated with development of asthma in children.	[10], [59]
*Prevotella melaninogenica*	Bacteroidetes	Neg	Commensal bacteria associated with the airway microbiota of healthy individuals.	[10]
*Prevotella nanceiensis*	Bacteroidetes	Neg	Commensal bacteria associated with the airway microbiota of healthy individuals.	[10]
*Prevotella salivae*	Bacteroidetes	Neg	Commensal bacteria associated with the airway microbiota of healthy individuals.	[10]
*Veillonella dispar*	Firmicutes	Neg	The most predominant commensal bacteria associated with the airway microbiota.	[10]
*Actinomyces graevenitzii*	Actinobacteria	Pos	Less predominant commensal bacteria associated with the airway microbiota.	[10]
*Actinomyces oris*	Actinobacteria	Pos	Less predominant commensal bacteria associated with the airway microbiota.	[10]

*NT: Non-typeable.

### Various airway bacteria induce similar dendritic cell maturation

**Figure 1:**
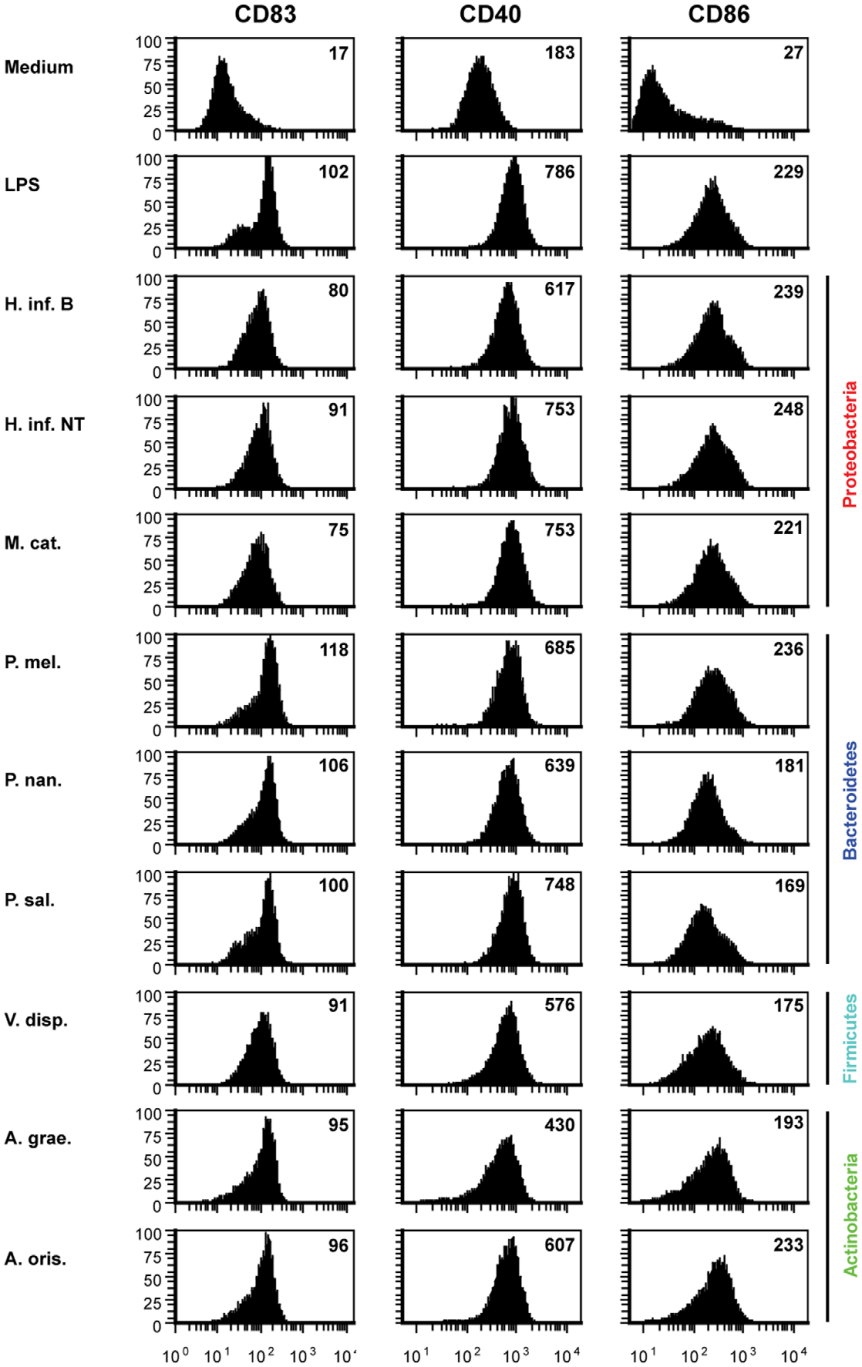
Maturation of dendritic cells stimulated with pathogenic and commensal airway bacteria. CD83, CD40 and CD86 expression by DCs in response to 24 h stimulation with medium, LPS, *Haemophilus influenzae* B (H. inf. B), non-typeable *Haemophilus influenzae* (H. inf. NT), *Moraxella catarrhalis* (M. cat.), *Prevotella melaninogenica* (P. mel.), *Prevotella nanceiensis* (P. nan.), *Prevotella salivae* (P. sal.), *Veillonella dispar* (V. disp.), *Actinomyces graevenitzii* (A. grae.) or *Actinomyces oris* (A. oris.). DCs were gated on viable cells. The geometric mean fluorescence intensity of the indicated marker is given on each chart. Data represents the response from one donor out of two.

Dendritic cells patrol peripheral tissues sampling the environment to sense invading microorganisms by recognizing microbial-associated molecular patterns or danger signals derived from tissues. Upon encounter with immunogenic components DCs acquire a mature phenotype enabling migration to nearby lymph nodes, crosstalk with T cells, and initiation of adaptive immune responses [12], [14]. CD83 is a well established differentiation marker used as a general indicator of DC maturation/activation *in vitro* [18], [19]. Furthermore, mature DCs upregulate the co-stimulatory markers CD40 and CD86 of importance for initiation and propagation of T cell activation via DC/T cell crosstalk. Here we used the CD83, CD40 and CD86 markers to address the presence of immune activating entities in both pathogenic and commensal bacteria. It was found, that all analyzed bacteria induced expression of these markers (figure 1) when compared to immature DC (medium). Lipopolysaccharide (LPS) recognized to promote DC maturation via TLR4 served as a positive control. The level of CD83, CD40 and CD86 expression between each bacterium were comparable suggesting that the bacteria had similar capability to induce DC maturation.

### Differential cytokine production by airway bacteria in dendritic cells

Mature dendritic cells produce cytokines that mediate inflammation and instruct development of antigen-specific helper T cells. IL-12p70 and IL-23 cytokines play a central role in mediating development or proliferation of Th1 and Th17 cells, respectively. Furthermore, these cytokines drive inflammation by stimulating production of the pro-inflammatory cytokines IFNγ (Th1) and IL-17 (Th17) by T cells leading to recruitment and activation of pro-inflammatory immune cells [20], [21]. On the contrary, IL-10 exhibit anti-inflammatory properties by inhibiting production of pro-inflammatory cytokines by various immune cells, including T cells, macrophages and epithelial cells [22]. In order to investigate the potential pro-inflammatory and anti-inflammatory properties of the pathogenic and commensal airway-associated bacteria, we analyzed the production of IL-23, IL-12p70 and IL-10 by the stimulated DCs. For each bacterium, we found that the average level of IL-23 and IL-10 cytokine production by DCs was generally 2–3 fold higher than the level of IL-12p70 (Table 2).

**Table 2:** Average cytokine production by DCs among all donors in response to bacteria or control stimuli.

Bacterial strain	Phylum	IL-23 (pg/ml)	IL-12p70 (pg/ml)	IL-10 (pg/ml)
Control	n/a	262	73	144
LPS	n/a	1829	1090	5500
*Haemophilus influenzae B*	Proteobacteria	11450	4424	12452
*Haemophilus influenzae NT* *	Proteobacteria	14108	5710	14797
*Moraxella catarrhalis*	Proteobacteria	13256	5507	14176
*Prevotella melaninogenica*	Bacteroidetes	2862	2684	6436
*Prevotella nanceiensis*	Bacteroidetes	2976	1751	7378
*Prevotella salivae*	Bacteroidetes	5267	2011	9869
*Veillonella dispar*	Firmicutes	5331	2450	5167
*Actinomyces graevenitzii*	Actinobacteria	156	81	1849
*Actinomyces oris*	Actinobacteria	147	36	1649

*NT: Non-typeable.

**Figure 2:**
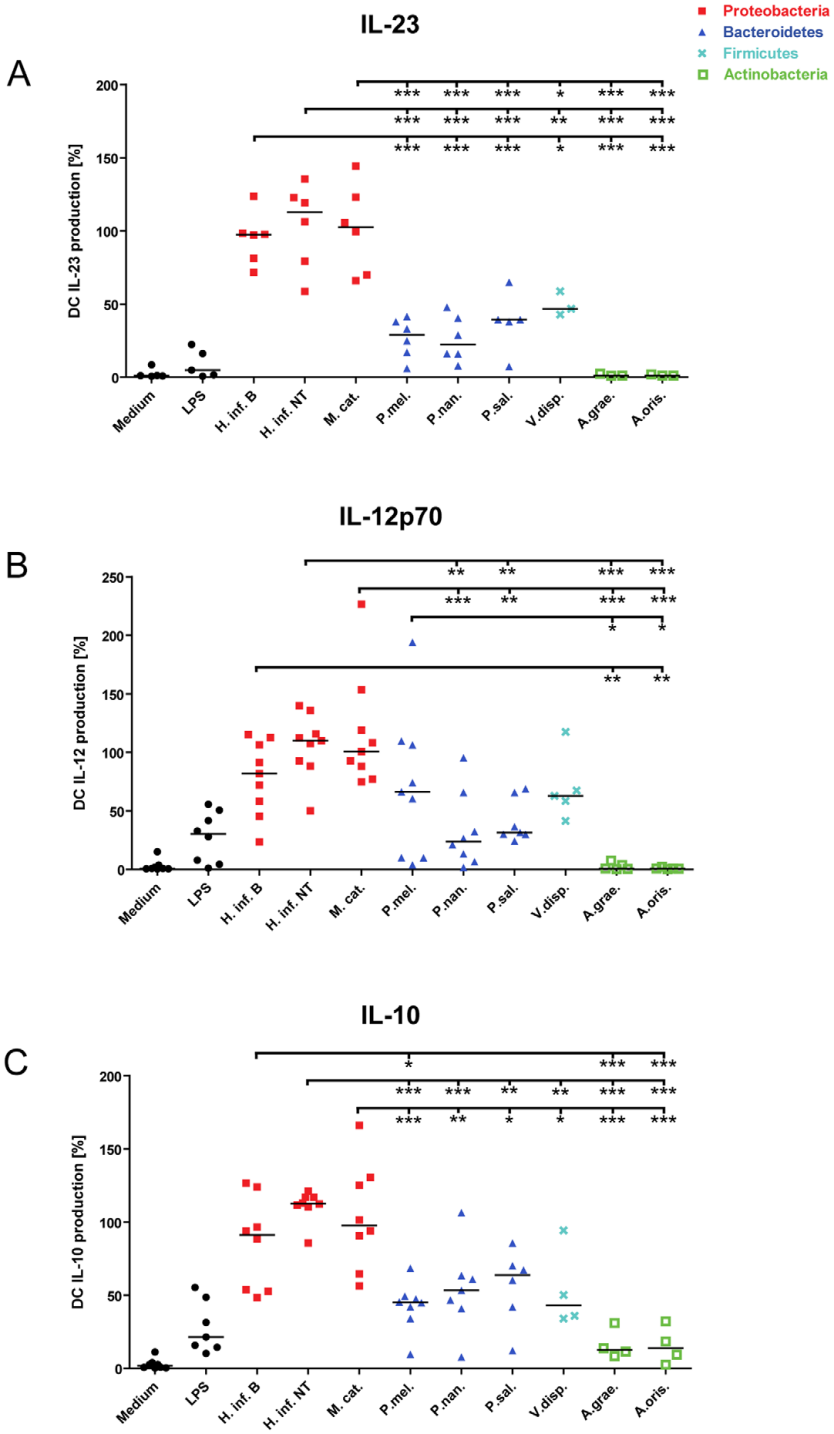
Cytokine production by dendritic cells stimulated with pathogenic and commensal airway bacteria. IL-23, IL-12p70 and IL-10 cytokine production measured in DC culture supernatants following 24 h stimulation with medium, LPS, *Haemophilus influenzae* B (H. inf. B), non-typeable *Haemophilus influenzae* (H. inf. NT), *Moraxella catarrhalis* (M. cat.), *Prevotella melaninogenica* (P. mel.), *Prevotella nanceiensis* (P. nan.), *Prevotella salivae* (P. sal.), *Veillonella dispar* (V. disp.), *Actinomyces graevenitzii* (A. grae.) or *Actinomyces oris* (A. oris.). Labels within each group represent different donors (n = 3–9). Cytokine production measurements were normalized to account for donor-specific variation. *p<0.05, **p<0.01, ***p<0.001.

Due to donor-specific variation, DC cytokine production was normalized to the average response of the three pathogenic bacteria, as these consistently induced similar and the highest cytokine production levels within each donor. Figure 2 show normalized cytokine production by DCs in response to the pathogenic and commensal bacteria. It was found that bacteria within the same family (*Haemophilus* spp., *Prevotella* spp. and *Actinomyces* spp.) and the corresponding phyla (proteobacteria, bacteriodetes, actinobacteria) induced similar levels of IL-23, IL-12p70 and IL-10. In general, pathogenic proteobacteria produced 3–5 fold higher levels of IL-23, IL-12p70 and IL-10 compared to the commensal bacteria. Lowest were the production of IL-23 and IL-12p70 by *Actinomyces* spp., which induced levels comparable to that of immature DCs.

### Segregation of airway bacteria into functional subgroups based on their inflammatory properties

**Figure 3:**
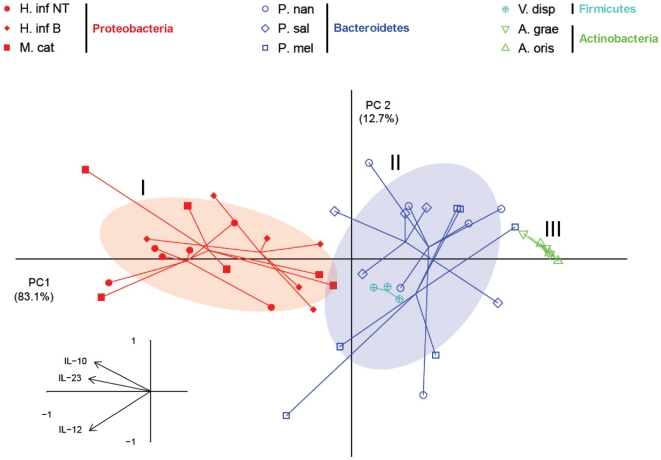
Principal component analysis of dendritic cell cytokine profiles in response to pathogenic and commensal airway bacteria. Principal component analysis reveals clustering of DC responses to airway bacteria. Three significantly different groups are distinguished by the production of IL-23, IL-12p70 and IL-10 cytokines. I: Pathogenic Gram-negative *Haemophilus influenzae* B (H. inf. B), non-typeable *Haemophilus influenzae* (H. inf. NT) and *Moraxella catarrhalis* (M. cat.); II: Commensal Gram-negative *Prevotella melaninogenica* (P. mel.), *Prevotella nanceiensis* (P. nan.), *Prevotella salivae* (P. sal.) and *Veillonella dispar* (V. disp.); III: Commensal Gram-positive *Actinomyces graevenitzii* (A. grae.) and *Actinomyces oris* (A. oris.). Responses to each bacterium were based on 3–6 different donors. Shaded areas represent the 67% confidence area within the three bacteria groups.

By use of a principal component analysis (PCA), it was possible to separate the bacteria species into three functionally distinct groups based on the levels of IL-23, IL-12p70 and IL-10 induced in DCs (figure 3). The PC1 and PC2 of the PCA score plot shown in figure 3 were found to describe more than 95% of the variance between the bacteria-induced DC responses. As indicated by the loading plot, the differences in levels of all cytokines (IL-23, IL-12p70 and IL-10) were the main factor responsible for discriminating the bacteria along PC1. The PCA analysis indicated that the bacteria could be divided into three groups: Highly stimulatory bacteria (Group I; *Haemophilus* spp. and *Moraxella* spp.), intermediately stimulatory bacteria (Group II; *Prevotella* spp. and *Veillonella* spp.), and weakly stimulatory bacteria (Group III; *Actinomyces* spp.). Using multivariate ANOVA it was found that the cytokine production profile were statistically significantly different between the identified groups (Group I vs. Group II: p<5.8 * 10^−10^; Group I vs. Group III: p<2.5* 10^−08^; Group II vs. Group III: p<0.00034). These results imply that it is possible to classify bacteria of the airway microbiota into distinct groups based on their functional immune profiles in DC that reflect properties of being associated with asthma and COPD, or healthy lungs.

### *Prevotella* strains reduce *Haemophilus*-induced IL-12p70 production by DC

**Figure 4:**
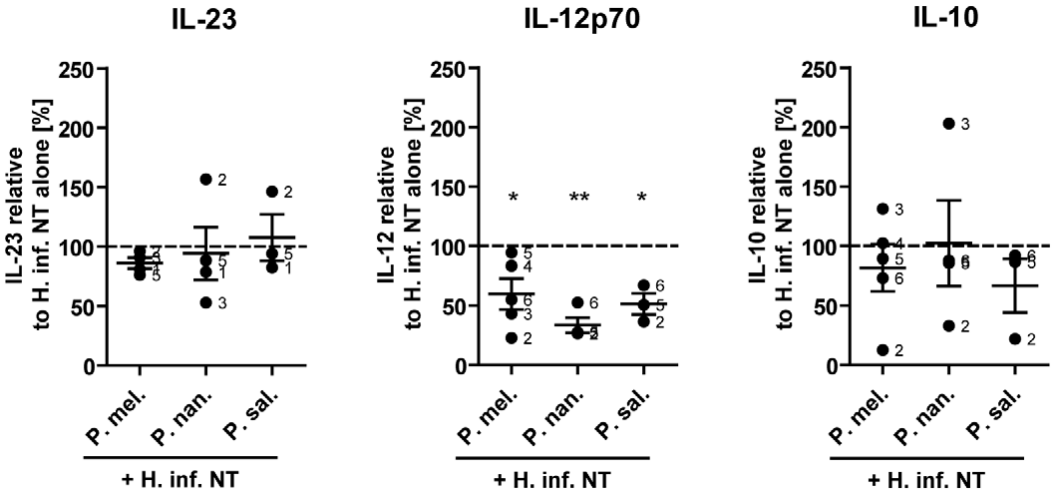
Modulation of *Haemophilus*-induced cytokine production in dendritic cells by *Prevotella* species. IL-23, IL-12p70 and IL-10 cytokine production measured in DC culture supernatants 24 h following stimulation with non-typeable *Haemophilus influenzae* (H. inf. NT) in combination with *Prevotella melaninogenica* (P. mel.), *Prevotella nanceiensis* (P. nan.) or *Prevotella salivae* (P. sal.). The effect of *Prevotella* species on *Haemophilus*-induced cytokine production was calculated relative to *Haemophilus* stimulation alone for each donor. The numbers identify individual donors (n = 3–5). *p<0.05, **p<0.01.

When present in tissues, bacteria with distinct functional characteristics are likely to interact simultaneously with the immune system affecting the overall response. This has been studied and demonstrated in relation to gut bacteria that modulated immune responses to other bacteria or MAMPs [23]. Yet it remains unknown if bacterial species associated with the airway microbiota demonstrate similar properties. Since *Haemophilus influenzae* is the pathogen most strongly associated with asthma and COPD [10], we analyzed the ability of three different *Prevotella* strains to modulate *Haemophilus*-induced IL-23, IL-12p70 and IL-10 production in DCs. It was found, that all studied *Prevotella* strains could partially reduce *Haemophilus*-induced IL-12p70 production by DCs, whereas no statistically significant effect on IL-23 and IL-10 production was apparent (figure 4). This indicates that commensal bacteria of the airways, similarly to the gut, may exhibit properties that enable modulation of the immune responses to specific pathogenic bacteria.

## Discussion

The commensal microbiota of the gastro-intestinal tract has demonstrated importance for metabolism, maturation of the immune system and protection from invasive microorganisms [24]. Furthermore, studies indicate that changes in the gut microbial composition are associated with inflammatory bowel disease [25], [26], with a protective role of specific commensal bacteria [27], [28]. Only recently has the existence of a commensal microbiota in the lower airways been appreciated and characterized [8]–[10]. The potential physiological role of these commensal airway bacteria remains to be established. To date, no studies have been reported addressing or comparing the immunological properties of the airway-associated commensal bacteria included in this study. Some studies have focused on the immunological mechanisms elicited by epithelial cells, macrophages and T cells in response to the pathogenic bacteria *Haemophillus influenzae* or *Moraxella catarrhalis* [29]–[34]. However, the response by DCs to these central respiratory pathogens remains to be clarified.

We found that all airway pathogenic and commensal bacteria included in our study induced similar maturation of DCs. This demonstrates that all the bacteria exhibit innate activating properties that may enable bacteria-exposed DCs to prime T cell responses. On the contrary to DC maturation, the bacteria induced varied levels of the T cell-polarizing cytokines IL-23, IL-12p70 and IL-10. This indicates that the bacteria may elicit different immunological processes *in vivo* due to the development of distinct helper T cell responses. The bacteria-specific differences in DC cytokine profiles are likely a result of the strain-specific composition of microbial-associated molecular patterns (MAMPs) that stimulate several innate receptors, including toll-like receptors (TLRs), NOD-like receptors (NLRs) and C-type lectin receptors (CLRs). Most strikingly is the absence of IL-23 and IL-12p70 in DCs stimulated with the commensal Gram-positive *Actinomyces* spp. when compared to the remaining airway bacteria that are all Gram-negative in origin. This difference is most likely based on the fact that cell membranes of Gram-positive bacteria lack LPS, contrarily to those of Gram-negative bacteria. LPS, especially in the presence of other MAMPs, is an effective inducer of IL-12p70 production in DCs via TLR4 [35]. Indeed, Gram-negative bacteria in general have been shown to induce more potent innate immune responses as compared to Gram-positive bacteria in a TLR4-dependent manner [36].

Within the group of Gram-negative bacteria (*Haemophilus* spp., *Moraxella* spp., *Prevotella* spp. and *Veillonella* spp.), we observed significantly higher levels of IL-23, IL-12 and IL-10 from DCs in response to the pathogenic bacteria species, *Haemophilus* spp. and *Moraxella* spp. The reason for the differences in DC cytokine-inducing properties amongst the Gram-negative airway bacteria could be the structure of LPS, which have been reported to vary amongst Gram-negative bacteria [37]. Particularly the structure of lipid A within LPS seems predominantly important for the biological activity. Notably, the classical LPS of E. coli is composed of hexa-acylated lipid A and seems to be the most biologically potent LPS structure [38]. However, some Gram-negative bacteria are reported to contain less potent atypical LPS composed of lipid A with shorter or fewer acyl chains [39]–[41]. LPS derived from *Haemophillus influenzae* and *Moraxella catarrhalis* has been reported to contain hexa- and hepta-acylated lipid A, respectively [42], [43]. No studies have been reported on LPS of the *Prevotella* or *Veillonella* species included in our study; yet LPS from other species within these genera have been analyzed. LPS of the oral commensal *Prevotella intermedia* contain penta-acylated lipid A and demonstrate about 10-fold reduced potency compared to LPS from E. coli [44]. Similarly, approximately 10-fold reduced potency in LPS has been reported from the gut commensal *Veillonella parvula* suggesting the absence of an optimal number of acyl chains in lipid A for propagation of a strong pro-inflammatory signal [45]. Combined these studies indicate, that the difference observed in this study between Gram-negative pathogenic and commensal bacteria of the airways may be due to varying potencies within their LPS components. However it should be noticed, that the pathogenic and commensal bacteria in this study are of different genera and other MAMPs within the bacteria could play a role in DC modulation via simultaneous engaging of receptors with pro- or anti-inflammatory activity.

In this study, we used monocyte-derived DCs to examine the bacteria-DC interaction as the quantity of DCs available per donor allowed us to screen for effects of several airway bacteria within the same donor. It should be emphasized that DCs of the airways, the cellular subset that will interact with airway microbes under in situ conditions, might respond differently than monocyte-derived DCs to the microbes. However, as DCs of the conducting airways and monocyte-derived DCs propagate from the common myeloid progenitor [46], it is likely that they share common response patterns. Airway DCs are known to sample environmental antigens in the airway lumen, and will likely be exposed to the collective bacterial ecosystem of the airway tract. It is therefore also possible that airway commensal bacteria may affect the overall DC response to potentially pathogenic bacteria. For this reason we studied how commensal *Prevotella* spp. affected the DC response to non-typeable *Haemophillus influenza* associated with asthma and COPD. We found that all *Prevotella* spp. reduced *Haemophillus influenza*-induced IL-12p70 production in DCs by approximate 50%, but had no effect on IL-23 and IL-10 levels. The explanation for this effect may relate to the structural differences in LPS between these species. Structurally atypical tetra- or penta-acylated LPS have been show to inhibit the more potent inflammatory response mediated by the classical hexa-acylated LPS via a process involving competition for MD2-binding [47]–[49]. While classical hexa-acylated LPS-MD2 dimerization results in TLR4-activation, the atypical LPS structures are suggested to affect TLR4-signaling by sequestering MD2 molecules. Consequently, the deficit in MD2 molecules needed for binding to classical LPS to initiate TLR4-signalling will result in reduced TLR4-activation, and thus lowered IL-12p70 levels. Studies in DCs indicate that TLR4 signaling by LPS favors IL-12p70 production by inducing the expression of IL-12p35 [50], whereas TLR2 ligands typically are poor IL-12p70 inducers. Rather, stimulation with TLR2 ligands in combination with NLR or dectin-1 ligands leads to IL-23 production in DCs by the preferential expression of IL-23p19 [51]. Combined, our present results suggest that the LPS of *Prevotella* spp., or other components present within this bacteria spp., inhibit the ability of *Haemophillus influenza* LPS to elicit complete TLR4 signaling hence leading to the reduction in IL-12p70 production by DCs. Yet, the presence of several TLR2 and NLR ligands within the complex bacteria may well allow for full level expression of IL-23. The biological relevance of IL-12p70 modulation in DCs by commensal bacteria remains elusive. However, it can be speculated that a change in the IL-23/IL-12 balance will translate into a predominant type-17 mediated responses by the DCs as IL-23 is the key mediator of proliferation and cytokine production by Th17 cells [52]. The modulation could enhance Th17 cell mediated immunity, which has been show to play a central role in clearance of pathogenic airway bacteria [53], [54].

The present study was primarily initiated due to the observation that commensal *Prevotella* spp. were absent, whereas pathogenic *Haemophillus* spp. and *Moraxella* spp. colonized the airways of asthmatics and COPD patients [10]. This finding suggests a divergent role of airway bacteria in chronic inflammatory airway diseases with a protective or modulator role in disease development. The association between pathogenic proteobacteria airway bacteria and COPD has been investigated for several decades giving rise to some controversy [55]. Yet, it is now established that pathogenic airway bacteria are associated with acute disease exacerbations, which leads to significant morbidity and mortality within this patient group [56], [57]. Pathogenic proteobacteria has more recently been associated with asthma in case-control studies [10], during exacerbation attacks [58], and reported as a risk factor for asthma development in children [59]. Interestingly, COPD, asthma exacerbations and some asthma phenotypes are associated with neutrophilic airway inflammation [60], [61], [56]. Th17 cells producing IL-17 are central mediators of neutrophil recruitment and activation in tissues, and shown to play a role in clearance of Gram-negative extracellular pulmonary pathogens [21]. This inflammatory pathway are primed and driven by IL-23 production by dendritic cells. In this study we found that pathogenic airway bacteria were potent inducers of IL-23 and IL-12p70 in DCs suggesting the development of bacteria-specific Th17 and Th1 cells *in vivo*. Studies in humans have demonstrated the development of a Th1 response to non-typeable *Haemophillus influenzae* [33], but the involvement of Th17 cells *in vivo* remains to be investigated. Yet some studies have reported neutrophilic airway inflammation, that share features with COPD and asthmatic inflammation, after airway installation of non-typeable *Haemophillus influenzae* in mice [62], [63]. This suggests the involvement of Th17 mediated inflammation in the response to pathogenic airway bacteria. It remains an enigma why pathogenic airway bacteria would induce potent inflammation that may drive their own clearance. However it could be speculated, that the potent inflammatory properties of pathogenic bacteria in combination with chronic colonization would translate into low-grade inflammation in tissues contributing to disease progression in both COPD and asthma.

## Materials and Methods

### Bacteria growth and preparation

*Haemophilus influenzae* B (KAK510), *Haemophilus influenzae* NT (KAK509) and *Moraxella catarrhalis* F48 (KAK508) reference stains were kindly provided by Karen Krogfelt and Jørgen Skov Jensen, Statens Serum Institut, Copenhagen, Denmark. *Prevotella melaninogenica* (DSM7089), *Prevotella nanceiensis* (DSM19126), *Prevotella salivae* (DSM15606), *Veillonella dispar* (DSM20735), *Actinomyces graevenitzii* (DSM15540), *Actinomyces oris* (DSM23056) were obtained from Deutsche Sammlung von Mikroorganismen und Zellkulturen (DSMZ), Braunschweig, Germany. *Haemophilus* and *Moraxella* strains were grown on chocolate agar plates (Statens Serum Institut) under 37°C microaerobic (5% CO_2_) conditions. *Prevotella* strains were grown on anaerobic agar plates (Statens Serum Institut) under 30°C anaerobic conditions. *Veillonella dispar* was grown on anaerobic agar plates under 37°C anaerobic conditions. *Actinomyces* strains were grown on chocolate agar plates under 30°C aerobic conditions. All strains were resuspended from plates with uniform growth and washed once in PBS. Bacteria were resuspended in PBS to OD 1 and UV-irradiated for 45 minutes. UV killing were confirmed by plating. Dry weights of bacteria suspensions in PBS were determined on 3×1 ml portions after freeze-drying (subtracted by weight of PBS). Bacterial suspensions were frozen and stored at −80°C.

### Dendritic cell preparation

Buffy coats prepared freshly from healthy anonymous blood donors were kindly provided by Rigshospitalet's Blood Bank, Copenhagen, Denmark. A total of 10 donors were used and they were picked randomly among eligible adult blood donors by blood bank staff. Experiments were performed at different days using one donor per day. Buffy coats were diluted 3-fold in complete RPMI 1640 medium (Lonza, Basel, Switzerland) supplemented with 2 mM L-glutamine (Cambrex, East Rutherford, NJ) and 100 U/ml penicillin/streptomycin (Lonza). PBMCs were isolated from diluted buffy coats by density centrifugation on Ficoll-Paque (Amersham Biosciences, Uppsala, Sweden). Monocytes were isolated from PBMCs using a CD14+ magnetic cell sorting kit (MACS; Miltenyi Biotec, Bergisch Gladbach, Germany) according to manufacturer's recommendations. Monocytes were differentiated into dendritic cells by plating 6*10^5^ cells/well (48-well plates) in 500 µl completed RPMI 1640 medium containing 10% FCS (Lonza), 30 ng/ml rhIL-4 and 20 ng/ml rhGM-CSF (both cytokines from Cellgenix, Freiburg, Germany) at day 0. Cytokines were refreshed on day 3, and fully differentiated dendritic cells were used at day 6. Differentiation of CD14^+^ monocytes into CD14^−^CD1a^+^ DCs was confirmed in our assay using flow cytometry.

### Dendritic cell stimulation and analysis

Dendritic cells were stimulated by replacing medium with complete RPMI 1640 medium containing stimuli supplemented with 50 µg/ml gentamycin (Sigma-Aldrich, Copenhagen, Denmark) to ensure no bacterial outgrowth. In preliminary experiments, the gentamycin at 50 µg/ml was found not to affect LPS-induced activation of DCs. All stimulations were done in triplicates, and a concentration of 50 µg/ml was used for all bacterial stimulations. In experiments with mixtures of two bacteria the concentration of each bacteria was 50 µg/ml. LPS (100 ng/ml) and medium alone were included to serve as a positive and negative control, respectively.

24 h after stimulation at 37°C, 5% CO_2_, DC supernatants were collected and stored at −20°C until analyses. Cytokines were measured in supernatants using commercial ELISA kits (IL-23; eBioscience, San Diego, CA; IL-12p70 and IL-10; R&D Systems, Minneapolis, MN) according to manufacturer's recommendations. In some experiments, DC surface expression of CD83, CD40 and CD86 were measured by flow cytometry. Briefly, 2*10^5^ cells were stained with fluorescein isothiocyanate-conjugated anti-hCD83, anti-hCD40 or anti-hCD86 (all from BD Pharmingen, San Jose, CA) in PBS containing 1% FCS and 0.1% sodium azide for 30 minutes at 4°C. Cells were analyzed on a BD FACSCanto™ II system running FACSdiva 6.0 software (BD Biosciences, San Jose, CA) followed by data analysis in FCS Express v4 (De Novo, Los Angeles, CA).

### Data analysis and statistics

Univariate statistical analysis was performed using GraphPad PRISM 5.01 (GraphPad Software, La Jolla, CA). Differences in normalized cytokine production were analyzed by one-way ANOVA with Tukey's multiple comparison test. *Prevotella* strains effect on *Haemophilus*-induced cytokine production in DCs were analyzed using Student's one sample t-test. Principal component analysis and multivariate ANOVA (MANOVA) analysis used to compare groups of bacteria were performed using the R software package (Foundation for Statistical Computing, Vienna, Austria). The PCA was performed with the prcomp function that uses Singular Value Decomposition on the covariance matrix for the PCA computations. P-values below 0.05 were considered statistically significant.
